# Comparative analysis of low-abrasive powders on root surface roughness and substance loss: an ex vivo study

**DOI:** 10.1007/s00784-026-07007-2

**Published:** 2026-07-17

**Authors:** Charlotte Wetzel, Ramona Hieble, Caspar Victor Bumm, Vasil Christoff, Andreas Kessler, Vinay Pitchika, Philipp Altpeter, Marcel Reymus, Falk Schwendicke, Matthias Folwaczny, Nils Werner

**Affiliations:** 1 Department of Conservative Dentistry, Periodontology and Digital Dentistry, LMU University Hospital, LMU Medizin, Ludwig-Maximilians-Universität München, Goethestraße 70, Munich, 80336 Germany; 2 Department of Prosthetic Dentistry, Center for Dental Medicine, Faculty of Medicine, Medical Center, University of Freiburg, Freiburg, Germany; 3 Faculty of Physics and Center for NanoScience (CeNS), Ludwig- Maximilians-Universität, Munich, Germany

**Keywords:** Air-Polishing,, Low-abrasive powders, Erythritol, Root surface topography, Subgingival instrumentation, Periodontitis

## Abstract

**Objective:**

To comparatively evaluate the effects of four low-abrasive air-polishing powders (glycine, erythritol, trehalose, and sodium bicarbonate) on root surface roughness and substance loss in an ex vivo model.

**Materials and methods:**

210 specimens from 105 human teeth were randomly allocated to four treatment groups (*n* = 50); 10 additional specimens served for measurement error analysis. Air-polishing was performed using glycine (25 μm), erythritol (14 μm), trehalose (30 μm), or sodium bicarbonate (40 μm). Three-dimensional surface topography was assessed by laser scanning microscopy before and after treatment using ISO 25,178 roughness parameters. Data were analyzed using rank-based ANCOVA with tooth type and surface location as covariates.

**Results:**

Post-treatment average roughness differed significantly between groups (*p* < 0.001, η^2^*p* = 0.217). Glycine (8.23 [5.45; 14.13] µm) and erythritol (6.65 [4.61; 10.22] µm) produced significantly higher values than trehalose (4.38 [2.87; 5.42] µm) and sodium bicarbonate (3.82 [2.83; 5.79] µm). Erythritol generated the most pronounced increases in valley depth and peak height. All groups showed negative skewness trends. No significant differences in substance loss were detected.

**Conclusion:**

Glycine and erythritol produced greater surface roughness than trehalose and sodium bicarbonate under the tested device configuration, while all powders demonstrated minimal substance loss, supporting their safety for subgingival application.

**Clinical relevance:**

Glycine and erythritol produced greater surface topographical changes compared to trehalose and sodium bicarbonate, which may reflect more pronounced powder-surface interaction; whether this translates into superior debridement efficacy requires direct microbiological investigation. The comparable and minimal substance loss across all powders supports their safety for repeated subgingival application.

**Supplementary Information:**

The online version contains supplementary material available at 10.1007/s00784-026-07007-2.

## Introduction

Air-polishing is considered an established, efficient, and gentle cleaning procedure for the removal of subgingival biofilm during active and supportive periodontal care. Compared to conventional mechanical instrumentation using hand or ultrasonic devices, air-polishing provides efficient biofilm removal with reduced treatment time, improved patient comfort and high clinical acceptance [1–3]. Modern air-powder-water systems allow precise control of pressure and water flow; specialized nozzles enable effective subgingival application with minimal tissue trauma.

For many years, large-particle sodium bicarbonate (200 μm) was the standard polishing powder for professional tooth cleaning. However, its high abrasiveness has been shown to cause excessive damage to root surfaces, particularly to the softer cementum and underlying dentin, resulting in deep surface defects, increased roughness (arithmetic average roughness parameter (Ra) increases of 3–5 μm), and substantial tissue loss (up to 200 μm in depth) compared to both untreated controls and gentler cleaning methods [4, 5]. As a result, current clinical guidelines recommend its use exclusively for supragingival enamel cleaning, while being contraindicated for subgingival use or application on exposed root surfaces [5]. In response, a range of low-abrasive powders with smaller particle sizes and lower abrasive potential, such as glycine, erythritol and trehalose, have been developed to enable safe and effective subgingival biofilm removal.

Glycine-based powders (25 μm) have demonstrated effective cleaning performance with excellent soft-tissue compatibility and minimal root surface damage [6]. Clinical studies have also demonstrated superior biofilm removal in pockets measuring 5–9 mm compared to ultrasonic instrumentation, particularly when used in conjunction with subgingival nozzles [7]. Glycine is also recommended for patients with exposed dentin or dentin hypersensitivity due to its gentle action and good biocompatibility, and it may support a more balanced subgingival microbiome [1].

Erythritol powder (14 μm) is characterized by an even smaller particle size, a lower abrasive effect, and increased density, which results in greater acceleration. It is well tolerated by patients and is perceived as more comfortable than traditional scaling procedures, contributing to its increasing use in professional cleaning and supportive periodontal care [3].

Trehalose powder (30 μm) represents a newer addition to low-abrasive polishing materials. With intermediate particle size between erythritol and glycine, it offers a gentle cleaning profile. A recent systematic review found comparable clinical outcomes with glycine and erythritol for probing depth reduction and bleeding on probing, suggesting similar efficacy and safety, though long-term data remain limited [8].

Despite the growing use of low-abrasive powders, little is known about their specific effects on root surface micromorphology. Since surface characteristics are critical for bacterial recolonization, tissue healing, and long-term tooth stability, understanding these effects is of major clinical relevance. Therefore, the aim of this ex vivo study was to investigate the effects of different low-abrasive powders routinely used for subgingival biofilm removal on the micromorphology of dental roots. 

### Methods

The study protocol was approved by the Ethics Committee of the Medical Faculty of Ludwig-Maximilians-University, Munich, Germany, with a positive vote granted on 09/26/2023 (Project-No. 23–0804). 

### Sample preparation

A total of 105 extracted, subgingival calculus- and caries-free human teeth were included in the investigation. Extractions were performed for periodontal reasons or based on strict medical indications. Immediately after extraction, the teeth were stored anonymously in a single collection container filled with Ringer’s solution. The maximum storage duration prior to the experiment was three months.

To obtain smooth and standardized surfaces, the teeth were gently cleaned under running water and subsequently sectioned longitudinally from the buccal to the oral side, to allow stable placement on the object table, using a precision saw (Isomet Low Speed, Buehler, Lake Bluff, IL, USA) equipped with a diamond-coated blade (LECO Instrumente GmbH, Mönchengladbach, Germany). In total, 210 tooth halves were obtained and individually stored in Ringer’s solution at 4 °C until further use. The samples were randomly assigned to four experimental groups, each consisting of 50 tooth halves, while an additional 10 specimens were reserved for the analysis of measurement error.

For standardized evaluation, each specimen was marked on the approximal surface 2 mm below the cementoenamel junction using a serrated handpiece saw blade. The marking consisted of a half-square outline, including only the upper edge and the left lateral border allowing consistent localization of the analysis area during both laser microscopy and powder application. The measurement area was defined prior to data analysis and held constant across all specimens; the central portion of the marked treatment zone was consistently selected to minimize positional sampling bias. 

### Sample size

Sample size was calculated a priori based on surface roughness data reported by Kruse et al. [9], who found roughness values of 0.371 ± 0.066 μm for air-polishing with erythritol on human dentin surfaces. For the four-group comparison (glycine, trehalose, erythritol, sodium bicarbonate) using one-way ANOVA, assuming a large effect size (Cohen’s f = 0.60), a significance level of α = 0.05, and a statistical power of 80%, a minimum sample size of 35 specimens per group (*n* = 140 total) was determined. Calculations were performed using G*Power (version 3.1). 

### Experimental setup

**Fig. 1:**
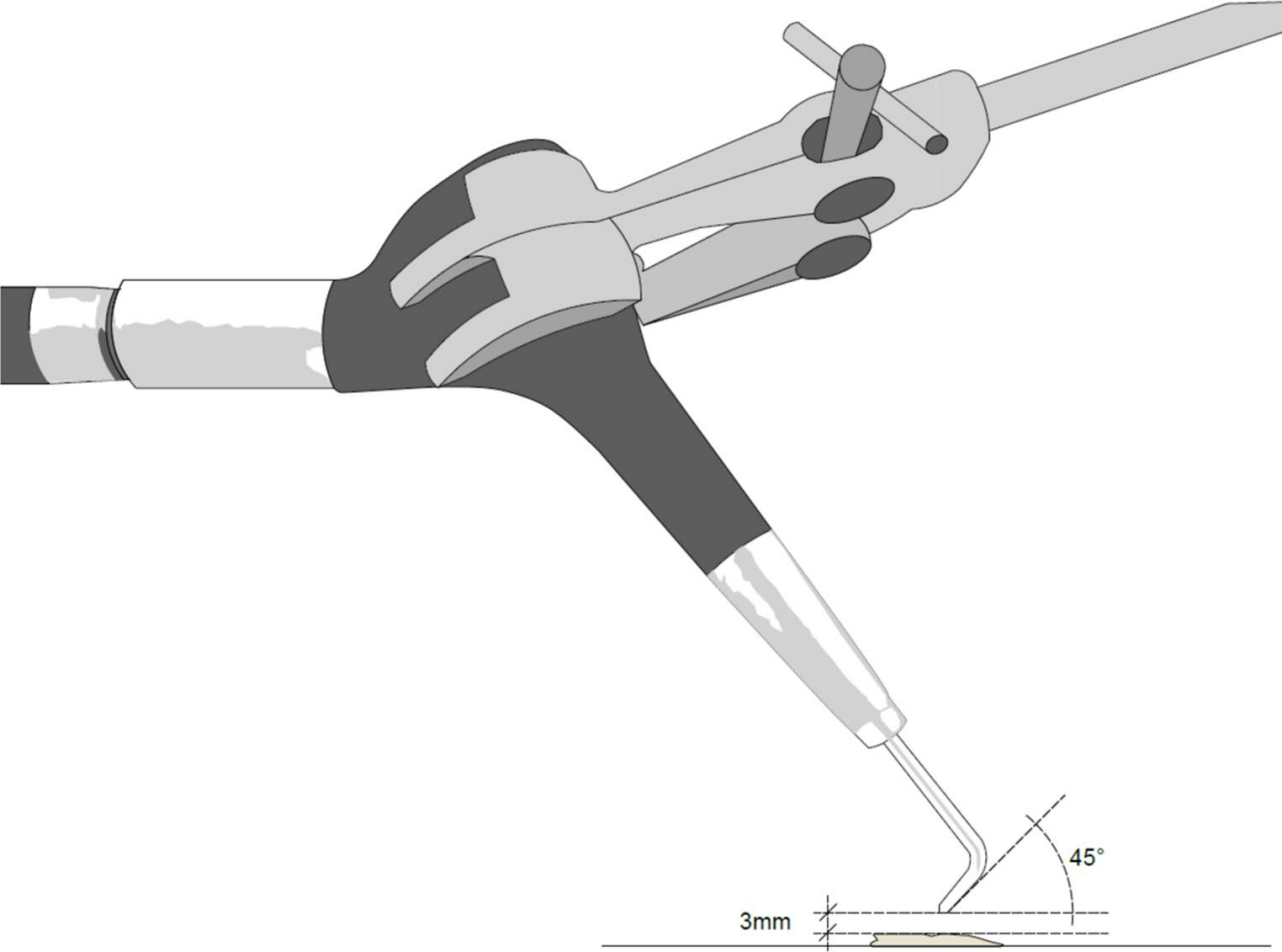
Schematic diagram of the experimental setup

Air-polishing was performed using a jet device (Prophyflex 4, KaVo, Biberach, Germany), mounted on a stand at a distance of 3 mm from the root surface and at an angle of 45° to the specimen (Fig. 1), as per clinical procedures and consistent with established ex vivo protocols [9, 10]. The previously marked area was treated for 15 s under static conditions, with the handpiece held at a fixed position throughout the application. To ensure consistent powder delivery, the powder chamber was refilled to its maximum capacity after each application.

The following powders were comparatively analysed:

- glycine (E.M.S. Electro Medical Systems S.A.) with a particle size of 25 μm,
- trehalose (Lunos, Dürr Dental SE) with a particle size of 30 μm,
- erythritol (E.M.S. Electro Medical Systems S.A.) with a particle size of 14 μm and.
- sodium bicarbonate (E.M.S. Electro Medical Systems S.A.) with a particle size of 40 μm.

**Fig. 2:**
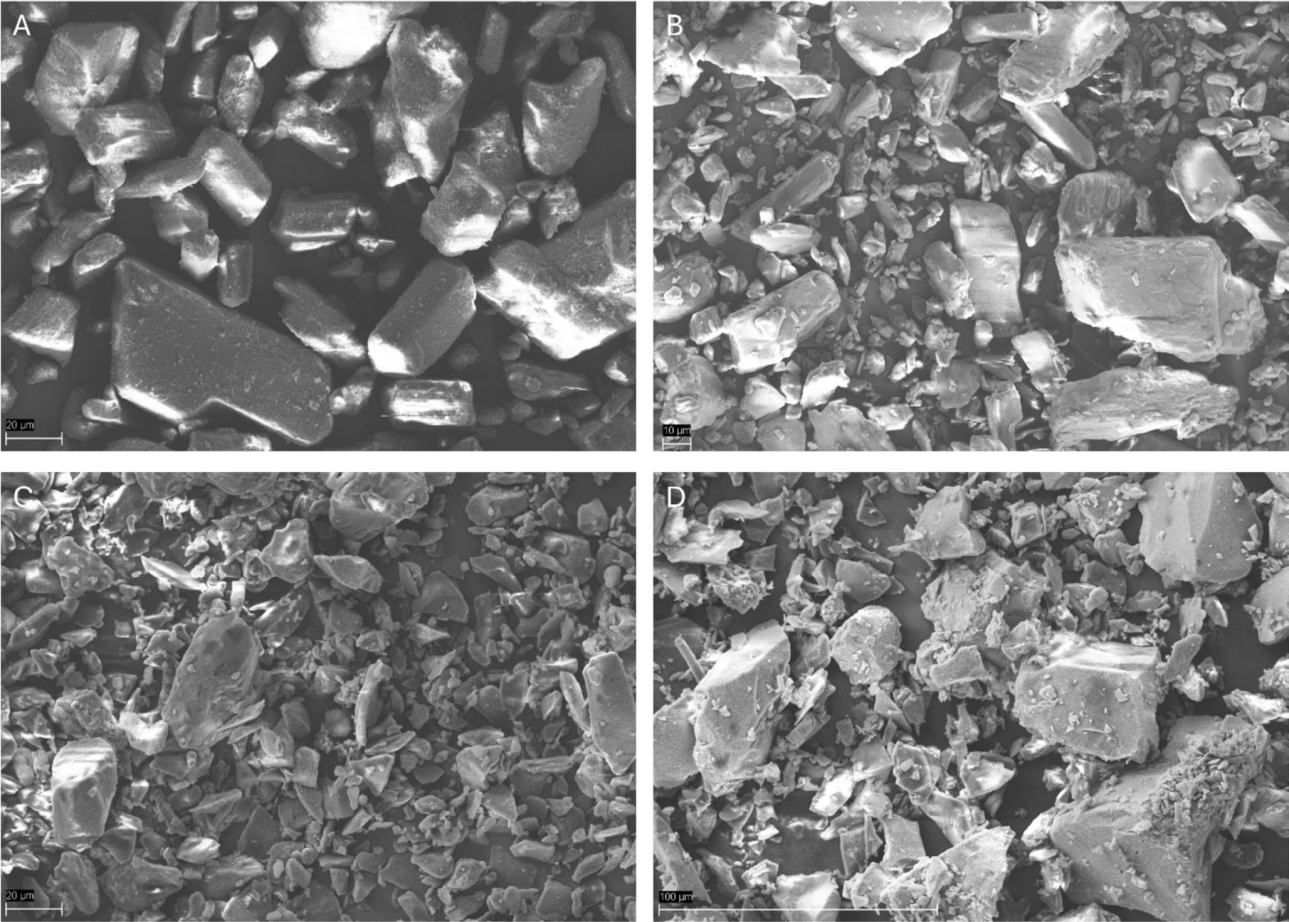
Scanning electron microscopy (SEM) image of sodium bicarbonate powder (**A**), glycine powder (**B**), erythritol (**C**), and trehalose (**D**) at 500× magnification

Representative scanning electron microscopy (SEM) images of the investigated powders at 500× magnification is shown in Fig. 2.

### Surface analysis

Prior to air-polishing, all specimens were examined using a laser-scanning microscope (Keyence VK-X3000, Osaka, Japan) to determine baseline surface roughness. Measurements were performed at 10× magnification with a lateral pitch of 6 μm. Each specimen was positioned so that the scan area aligned precisely with the predefined marking, allowing repeated measurements of the same region. A moderate-level 2 filter was applied to reduce speckle noise and minor sensor artifacts without altering relevant microstructural features. For standardized evaluation, an area of 800 μm × 250 μm was extracted for analysis, enabling nanometre-scale height resolution with micrometre-level lateral accuracy. After air-polishing, teeth were rinsed under running water and stored in Ringer’s solution for transportation for a maximum of three months prior to use. All specimens were rescanned using identical settings and specimen positioning to ensure direct comparability with baseline measurements.

Surface roughness parameters (Table 1) were analysed to assess changes in root surface topography following air-powder treatment. Mean substance loss (µm) was calculated using the compare function of the MultiFileAnalyzer software (Keyence, Osaka, Japan) by superimposing baseline and post-treatment scans and computing the mean height difference. Representative laser scanning microscopy images of root surface specimens before and after treatment are provided in Figure S1.

**Table 1:** ISO 25,178 Surface texture parameters: definitions and relevance for evaluating air-polishing effects on root surfaces

Parameter	Definition	Relevance for Root Surface Analysis
Sa (Arithmetical mean height) [µm]	Mean height deviation of the surface across the entire measurement area.	Indicates overall surface roughness; increase = greater roughening, decrease = smoothing effect.
Sz (Maximum height) [µm]	Vertical distance between the highest peak and deepest valley of the surface.	Sensitive to extreme peaks or deep scratches; important for detecting substance loss or powder-induced defects.
Str (Texture aspect ratio) [0–1]	Measure of surface texture uniformity; values near 1 = isotropic (uniform), values near 0 = anisotropic (directional).	Indicates whether powder treatment produces uniform or directional surface patterns; relevant for assessing homogeneous surface preservation.
Spc (Arithmetic mean peak curvature) [1/mm]	Mean curvature of surface peaks; high values = sharp peaks, low values = rounded peaks.	Indicates whether powder treatment creates sharp or blunted peak profiles; relevant for biofilm retention potential.
Sdr (Developed interfacial area ratio) [%]	Percentage increase in surface area compared to a perfectly flat reference plane.	Important factor for plaque adhesion and biofilm retention; higher values indicate greater surface complexity.
Ssk (Skewness) [dimensionless]	Asymmetry of the height distribution; negative values = valley-dominated surface, positive values = peak-dominated surface.	Indicates whether treatment creates new elevations (positive shift) or exposes/deepens valleys (negative shift); key parameter for interpreting cleaning depth.
Sp (Maximum peak height) [µm]	Height of the highest peak above the mean plane.	Detects isolated prominent peaks; relevant for assessing extreme surface protrusions.
Sv (Maximum pit height) [µm]	Depth of the deepest valley below the mean plane.	Detects deep defects (e.g., debris niches, substance loss); relevant for assessing maximum surface damage.

### Source of bias

All experimental procedures were performed by a single operator (RL), while all measurements were conducted by a separate examiner (NW). To minimize information bias, NW was blinded to the air-polishing allocation for each specimen. Prior to treatment, all teeth were examined under optical loupes to identify potential damage that could lead to exclusion, thereby reducing the risk of misclassification bias. For quality control of change in substance measurements, five untreated teeth and five teeth treated with aluminium oxide (positive control) were randomly interspersed among all specimens, revealing a systematic error of -0.7 μm and a maximum detectable substance change of -23.7 μm. Confounding bias was minimized by acquiring all scans consecutively under identical conditions in a controlled, clean-room environment, thereby avoiding variability introduced by storage conditions.

### Statistical analysis

Descriptive statistics are presented as median [Q1; Q3] due to non-normal distributions in several parameters. Surface roughness parameters were compared between four air-polishing treatment groups (glycine 25 μm, trehalose 30 μm, erythritol 14 μm, and sodium bicarbonate 40 μm) using analysis of covariance (ANCOVA) with tooth type (anterior, premolar, molar) and surface (mesial, distal) as covariates. Interaction effects (Group × Toothtype, Group × Surface) were tested using likelihood ratio tests but found to be non-significant (all false discovery rate (FDR)-corrected *p* > 0.05), supporting the use of the main effects model. Post-hoc pairwise comparisons were performed using estimated marginal means (EMMs) with FDR correction (Benjamini-Hochberg). Significance groups are indicated using compact letter display based on EMMs; groups sharing the same letter are not significantly different (*p* > 0.05). Effect sizes are reported as partial eta-squared (η^2^p), interpreted as: small (0.01), medium (0.06), and large (≥ 0.14). Baseline group homogeneity was assessed using Kruskal-Wallis tests. All analyses were performed using R version 4.5.1.

## Results

A total of 210 root surface specimens were analyzed (50 per treatment group, additional 10 for analysis of measurement error). The distribution of tooth types and surfaces is presented in Table 2. Across all groups, molars and premolars predominated, representing 72–84% of specimens. Mesial surfaces were slightly more frequent than distal surfaces in all treatment groups (52–68% vs. 32–48%).

**Table 2:** Sample characteristics by treatment group

Treatment Group		Tooth Type			Surface	
	*n*	Anterior *n* (%)	Premolar *n* (%)	Molar *n* (%)	Mesial *n* (%)	Distal *n* (%)
Trehalose 30 μm	50	8 (16.0)	14 (28.0)	28 (56.0)	26 (52.0)	24 (48.0)
Bicarbonate 40 μm	50	10 (20.0)	18 (36.0)	22 (44.0)	32 (64.0)	18 (36.0)
Erythritol 14 μm	50	20 (40.0)	14 (28.0)	16 (32.0)	32 (64.0)	18 (36.0)
Glycine 25 μm	50	13 (26.0)	20 (40.0)	17 (34.0)	34 (68.0)	16 (32.0)

The baseline surface parameters of the extracted teeth, prior to air-polish treatment, were comparable between the groups. No statistically significant differences were found in any of the parameters examined. This indicates that the surface structure at baseline was comparable across all groups (Table 3).

**Table 3:** Baseline surface roughness parameters by treatment group

Parameter	Trehalose 30 μm	Bicarbonate 40 μm	Erythritol 14 μm	Glycine 25 μm	*p*-value
Sa	3.89 [2.47; 5.31]	3.42 [2.45; 4.37]	3.79 [3.02; 4.87]	4.36 [2.62; 7.00]	0.092
Sz	51.88 [38.62; 67.56]	49.06 [37.99; 74.80]	59.40 [44.38; 82.61]	62.66 [41.07; 77.74]	0.223
Str	0.59 [0.40; 0.70]	0.54 [0.34; 0.68]	0.42 [0.30; 0.66]	0.49 [0.30; 0.68]	0.367
Spc	1233.12 [1027.92; 1549.39]	1202.26 [986.26; 1437.42]	1135.39 [907.18; 1479.74]	1172.86 [872.70; 1557.57]	0.333
Sdr	0.52 [0.32; 0.76]	0.45 [0.27; 0.74]	0.41 [0.28; 0.66]	0.48 [0.24; 0.76]	0.568
Ssk	0.59 [0.19; 1.50]	0.53 [0.06; 1.48]	0.55 [-0.26; 0.95]	0.31 [-0.36; 0.99]	0.155
Sp	45.01 [30.57; 76.33]	39.55 [29.04; 69.60]	35.36 [27.19; 51.42]	36.62 [27.14; 74.46]	0.236
Sv	29.44 [20.67; 46.49]	29.92 [22.22; 48.43]	30.53 [21.09; 45.45]	35.53 [19.92; 51.62]	0.868
Sal	101.58 [95.13; 118.19]	99.10 [82.76; 119.69]	99.38 [86.42; 120.83]	102.75 [86.14; 112.93]	0.705

Values are median [Q1; Q3]. p-values from Kruskal-Wallis test for baseline group differences

### Surface roughness changes following air-polishing treatment and substance loss

Changes in surface roughness parameters from baseline to post-treatment are presented in Table 4 and Figure S2. Most surface parameters showed no significant differences between powders, including ΔSa (arithmetical mean height), ΔSz (maximum height), ΔStr (texture aspect ratio), ΔSpc (arithmetic mean peak curvature), ΔSdr (developed interfacial area ratio), ΔSsk (skewness), and ΔSal (auto-correlation length) (all *p* > 0.05). However, significant group differences emerged for specific height distribution parameters. For ΔSp (maximum peak height), erythritol demonstrated a significantly higher increase (18.52 μm [0.84; 40.76]) compared to trehalose (-2.86 μm [-27.45; 7.02], *p* < 0.05), with sodium bicarbonate and glycine showing intermediate values not significantly different from either group. For ΔSv (maximum pit depth), erythritol produced significantly higher increases (19.24 μm [0.96; 47.24]) compared to all other powders (trehalose: -0.16 μm [-6.55; 5.15]; sodium bicarbonate: 2.39 μm [-7.23; 11.82]; glycine: 1.14 μm [-5.24; 10.76]; all *p* < 0.05).

**Table 4:** Surface roughness parameter changes by treatment group

Parameter	Trehalose 30 μm	Bicarbonate 40 μm	Erythritol 14 μm	Glycine 25 μm
ΔSa	-0.10 [-0.48; 1.33]^a^	0.49 [-0.15; 1.99]^a^	2.69 [0.11; 5.16]^a^	3.69 [1.19; 8.01]^a^
ΔSz	0.38 [-7.51; 7.80]_a_	0.75 [-11.73; 11.26]^a^	17.56 [-11.06; 54.24]^a^	14.92 [2.60; 32.07]^a^
ΔStr	-0.03 [-0.21; 0.06]_a_	-0.03 [-0.20; 0.09]^a^	-0.05 [-0.26; 0.03]^a^	-0.02 [-0.23; 0.15]^a^
ΔSpc	-74.53 [-265.88; 147.25]^a^	-59.82 [-306.14; 146.11] ^a^	-13.10 [-369.19; 233.68]^a^	-125.47 [-448.66; 86.22]^a^
ΔSdr	-0.11 [-0.30; 0.04]^a^	-0.03 [-0.27; 0.14]^a^	-0.05 [-0.26; 0.14]^a^	-0.06 [-0.40; 0.07]^a^
ΔSsk	-0.28 [-0.93; 0.22]^a^	-0.46 [-1.31; 0.20]^a^	-0.17 [-1.40; 0.47]^a^	-0.16 [-0.99; 0.29]^a^
ΔSp	-2.86 [-27.45; 7.02]^a^	-4.30 [-12.91; 16.38]^ab^	18.52 [0.84; 40.76]^b^	-4.46 [-22.47; 11.23]^ab^
ΔSv	-0.16 [-6.55; 5.15]^a^	2.39 [-7.23; 11.82]^a^	19.24 [0.96; 47.24]^b^	1.14 [-5.24; 10.76]^a^
ΔSal	8.43 [-5.24; 31.78]^a^	14.27 [-7.03; 37.46]^a^	13.95 [-5.14; 48.04]^a^	12.41 [-12.61; 33.07]^a^

Values are median [Q1; Q3]. Δ = post-treatment − baseline

Superscript letters indicate significance groups based on estimated marginal means (EMMs) adjusted for tooth type and surface; groups sharing the same letter are not significantly different (FDR-corrected *p* > 0.05)

Post-treatment surface roughness parameters and substance loss are presented in Table 5 and Figure S3. The primary roughness parameter Sa revealed significant differences between treatment groups (*p* < 0.05). Glycine produced the highest surface roughness (8.23 μm [5.45; 14.13]), followed by erythritol (6.65 μm [4.61; 10.22]), with both powders yielding significantly greater roughness than trehalose (4.38 μm [2.87; 5.42]) and sodium bicarbonate (3.82 μm [2.83; 5.79]). Trehalose and sodium bicarbonate did not differ significantly from each other.

**Table 5:** Post-treatment surface roughness parameters and substance loss by treatment group

Parameter	Trehalose 30 μm	Bicarbonate 40 μm	Erythritol 14 μm	Glycine 25 μm
Sa	4.38 [2.87; 5.42] a	3.82 [2.83; 5.79] a	6.65 [4.61; 10.22] b	8.23 [5.45; 14.13] c
Sal	115.34 [99.35; 136.23] a	117.90 [91.24; 135.77] a	118.18 [97.65; 146.97] a	108.45 [94.39; 136.47] a
Sdr	0.43 [0.32; 0.54] a	0.39 [0.25; 0.55] a	0.35 [0.25; 0.51] a	0.31 [0.19; 0.46] a
Sp	45.01 [31.53; 55.35] a	40.01 [23.80; 70.03] a	64.87 [40.39; 83.06] a	41.04 [26.19; 54.07] a
Spc	1179.60 [1077.42; 1342.91] a	1149.58 [983.87; 1341.20] a	1085.92 [968.37; 1296.41] a	1052.56 [867.02; 1190.37] a
Ssk	0.37 [-0.10; 0.78] a	-0.04 [-0.40; 0.43] a	-0.09 [-0.63; 0.67] a	-0.05 [-0.50; 0.43] a
Str	0.45 [0.33; 0.60] b	0.51 [0.26; 0.62] b	0.30 [0.26; 0.45] a	0.39 [0.27; 0.57] ab
Sv	30.23 [23.53; 45.61] a	31.26 [22.68; 53.23] a	49.82 [31.61; 79.87] b	39.96 [24.27; 58.19] a
Sz	50.69 [35.56; 80.10] a	49.82 [37.28; 74.24] ab	83.89 [63.77; 104.15] b	84.16 [59.04; 108.35] b
Change in substance	-12.992 ± 33.477 a	-7.746 ± 29.965 a	-2.343 ± 34.375 a	-6.153 ± 15.806 a

Values are median [Q1; Q3] unless otherwise stated. Substance Loss is reported as mean ± standard deviation (SD)

Superscript letters indicate significance groups based on estimated marginal means (EMMs) adjusted for tooth type and surface; groups sharing the same letter are not significantly different (FDR-corrected *p* > 0.05)

For maximum height parameters, Sz demonstrated significant group differences, with erythritol (83.89 μm [63.77; 104.15]) and glycine (84.16 μm [59.04; 108.35]) producing significantly greater values compared to trehalose (50.69 μm [35.56; 80.10], *p* < 0.05), while sodium bicarbonate showed intermediate values (49.82 μm [37.28; 74.24]). Similarly, Sv was significantly increased following erythritol treatment (49.82 μm [31.61; 79.87]) compared to all other powders (trehalose: 30.23 μm [23.53; 45.61]; sodium bicarbonate: 31.26 μm [22.68; 53.23]; glycine: 39.96 μm [24.27; 58.19]; all *p* < 0.05).

Str showed that erythritol produced significantly lower values (0.30 [0.26; 0.45]), indicating more directional surface patterns, compared to trehalose (0.45 [0.33; 0.60]) and sodium bicarbonate (0.51 [0.26; 0.62]), with glycine demonstrating intermediate characteristics (0.39 [0.27; 0.57]).

No significant differences were observed between treatment groups for Sal, Sdr, Spc or Ssk (all *p* > 0.05). Substance loss measurements revealed no significant differences between treatment groups (*p* > 0.05). 

### Treatment group effects on surface parameters

The rank-based ANCOVA, adjusted for tooth type and surface, revealed significant between-group differences in several surface texture parameters (Table 6). Among the change parameters, ΔSa showed the largest effect (F(3, 193) = 20.07, *p* < 0.001, η^2^*p* = 0.244), followed by ΔSp (F(3, 193) = 6.80, *p* < 0.001, η^2^*p* = 0.091), ΔSv (F(3, 193) = 8.14, *p* < 0.001, η^2^*p* = 0.089), and ΔSz (F(3, 193) = 5.73, *p* < 0.001, η^2^*p* = 0.085). Post-treatment values similarly differed significantly across groups for Sa (F(3, 193) = 16.63, *p* < 0.001, η^2^*p* = 0.217), Sz (F(3, 193) = 9.17, *p* < 0.001, η^2^*p* = 0.131), Sv (F(3, 193) = 6.16, *p* < 0.001, η^2^*p* = 0.082), Sp (F(3, 193) = 5.50, *p* = 0.003, η^2^*p* = 0.078), and Spc (F(3, 193) = 3.38, *p* = 0.041, η^2^*p* = 0.058).

**Table 6:** Rank-based ANCOVA results for treatment group effects

Variable	F (df)	*p*-value	*p* (FDR)	η^2^*p*	
ΔSa	20.07 (3, 193)	< 0.001	< 0.001	0.244	*
ΔSz	5.73 (3, 193)	< 0.001	0.002	0.085	*
ΔStr	0.33 (3, 193)	0.801	0.818	0.005	
ΔSpc	0.90 (3, 193)	0.442	0.600	0.015	
ΔSdr	0.58 (3, 193)	0.628	0.746	0.009	
ΔSsk	0.43 (3, 193)	0.731	0.817	0.007	
ΔSp	6.80 (3, 193)	< 0.001	< 0.001	0.091	*
ΔSv	8.14 (3, 193)	< 0.001	< 0.001	0.089	*
ΔSal	0.31 (3, 193)	0.818	0.818	0.003	
Sa (post)	16.63 (3, 193)	< 0.001	< 0.001	0.217	*
Sz (post)	9.17 (3, 193)	< 0.001	< 0.001	0.131	*
Str (post)	2.79 (3, 193)	0.042	0.079	0.045	
Spc (post)	3.38 (3, 193)	0.019	0.041	0.058	*
Sdr (post)	1.85 (3, 193)	0.140	0.204	0.036	
Ssk (post)	1.88 (3, 193)	0.134	0.204	0.024	
Sp (post)	5.50 (3, 193)	0.001	0.003	0.078	*
Sv (post)	6.16 (3, 193)	< 0.001	0.002	0.082	*
Sal (post)	0.77 (3, 193)	0.515	0.652	0.010	
Substance Loss	0.92 (3, 193)	0.431	0.611	0.017	

* *p* < 0.05 (FDR-corrected). Rank-based ANCOVA adjusted for tooth type and surface. η^2^p = partial eta-squared based on ranked data

In contrast, no significant differences were observed for spatial or functional parameters, including ΔStr, ΔSpc, ΔSdr, ΔSsk, ΔSal, and their post-treatment equivalents (all FDR-corrected *p* > 0.05). Notably, substance loss showed no significant differences between treatment groups with a negligible effect size (η^2^*p* = 0.017). These omnibus tests confirmed the pairwise comparisons reported in Tables 4 and 5, among the significant parameters, post-treatment Sa demonstrated the strongest discriminatory power between treatment groups (η^2^*p* = 0.217), followed by post-treatment Sz (η^2^*p* = 0.131), while among change scores, ΔSa showed the largest effect (η^2^*p* = 0.244), followed by ΔSp (η^2^*p* = 0.091) and ΔSv (η^2^*p* = 0.089).

## Discussion

The present ex vivo study investigated the effects of four different air-polishing powders on root surface roughness parameters and substance loss. Our results demonstrate significant differences in post-treatment surface roughness among the tested powders, while substance loss remained comparable across all groups. These findings contribute to the growing body of evidence regarding the safety and efficacy of modern low-abrasive air-polishing powders for professional tooth cleaning on exposed root surfaces.

In the present study, three-dimensional surface topography was analysed using a laser scanning microscope, enabling a highly detailed assessment with nanometre-scale vertical resolution and micrometre-level lateral precision. In contrast to several previous studies that examined flattened or sectioned specimens [5, 11], this investigation preserved the native curved root-surface architecture, which more accurately reflects clinical conditions. The software facilitates comparative measurements on curved surfaces with micrometre-level precision, a capability not available with conventional profilometers that require specimen flattening [9], however, the method is prone to errors: Laser scanning microscopy is susceptible to optical artifacts (speckle noise, surface reflections) and measurement errors on highly curved or steep surfaces due to angle-dependent light scattering. To validate the reliability of this measurement method, control specimens (five untreated and five treated with aluminium oxide) were randomly interspersed throughout the dataset, revealing a systematic measurement error of -0.7 μm, which is considered highly satisfactory and similar to values reported in recent metrology studies [9]. Nevertheless, the analysed area was relatively small (< 1 × 1 mm), which may limit a comprehensive assessment of surface heterogeneity across larger root surface areas. The selected area size represents a pragmatic compromise and is comparable to measurement parameters employed in similar investigations [4, 12].

To the best of our knowledge, this represents one of the first studies to comprehensively analyse three-dimensional ISO 25,178 surface parameters, including Sp, Sv, and Ssk, on root surfaces following air-polishing treatment. Previous investigations have primarily relied on conventional two-dimensional roughness parameters (Ra/Sa, average maximum height of the profile (Rz) /Sz), which may not fully capture the topographical changes induced by different air-polishing powders [4, 13]. Here, an increased surface roughness of 0.2 μm in Ra was associated with higher biofilm retention [14]; however, data for other surface parameters are not available, which raises questions about their clinical relevance. Other studies [15–17] applied ISO 25,178 parameters to composite surfaces, demonstrating that Ssk provides valuable information regarding surface symmetry alterations. Further, the maximum peak height (Sp) and maximum valley depth (Sv) parameters capture the extreme topographical features that may be most relevant for biofilm retention and tissue adaptation. Studies in implant dentistry have demonstrated that surface peaks can impede fibroblast attachment and epithelial migration, while deep valleys may serve as protected niches for bacterial colonization [18]. Research on biofilm formation has shown that surface valleys exceeding 10 μm in depth can harbor bacteria that are protected from shear forces and antimicrobial agents [14, 19]. Notably, the inclusion of these advanced parameters, combined with paired pre- and post-treatment measurements, may provide a more comprehensive characterization of powder-induced surface modifications, including subtle changes in peak-valley distribution and micro-scale substance loss that conventional amplitude parameters alone cannot detect. 

### Interpretation of roughness changes

Post-treatment Sa values demonstrated significant differences between treatment groups (*p* < 0.001, η^2^*p* = 0.217), with glycine and erythritol producing significantly higher average roughness than trehalose and sodium bicarbonate (*p* < 0.05). This hierarchy is not explained by particle size alone: the smallest particles (erythritol, 14 μm) did not yield the smoothest surfaces, and the largest (sodium bicarbonate, 40 μm) produced moderate rather than extreme roughness. Comparable findings have been reported for erythritol on dentin in controlled ex vivo settings [9], though direct comparisons across studies are limited by differences in substrate preparation and measurement methodology.

At first glance, higher post-treatment Sa values for glycine and erythritol appear to contradict the well-established understanding of sodium bicarbonate as the most abrasive of the tested powders [4, 12]. Three mechanistically distinct explanations are proposed.

First, the sodium bicarbonate powder used in this study had a particle size of 40 μm, substantially smaller than the historically used formulations of up to 200 to 250 μm that underlie the established abrasiveness literature and which produce Ra increases of 3–5 μm and substance loss of up to 200 μm on root surfaces [4, 5]. Abrasive potential is directly influenced by particle size, hardness and angularity, with larger particles generating proportionally greater surface damage [15]. Studies comparing larger sodium bicarbonate particles with glycine powders on root dentin have consistently shown greater roughness for sodium bicarbonate [20] - yet those findings employed particle sizes markedly larger than the 40 μm formulation tested here. When particle size is substantially reduced, the kinetic energy per impact is correspondingly attenuated, and the abrasive differential between powders narrows considerably.

Second, device configuration may have modulated inter-powder abrasive differences. The Prophyflex 4 device does not hold regulatory approval for all tested powders, and device-powder interactions - including particle velocity, spray pattern, and powder flow rate - can substantially affect the surface impact characteristics of individual powders [21]. Under a standardized device configuration, powders not optimized for that specific device may perform differently than their published physical properties would predict.

Third, specimens in the present study were derived from extracted teeth carrying residual surface deposits from the extraction and preparation process, rather than pristine, artificially prepared surfaces as employed in several reference studies [4, 12]. Under these conditions, the organic amino acid and polyol powders glycine and erythritol may interact more actively with proteinaceous and organic surface residues, producing greater topographical modification through a combination of mechanical impact and physicochemical surface interaction, rather than through only abrasive cutting of the underlying mineral substrate. However, this hypothesis has not been directly investigated and requires further investigations.

It is conceivable that powders with distinct particle dynamics engage deposit-bearing surfaces differently than clean cementum, potentially contributing to the observed roughness hierarchy. This speculative mechanism notwithstanding, the absence of significant differences in substance loss across all groups (*p* = 0.431, η^2^*p* = 0.017) and the confirmed baseline group homogeneity (*p* = 0.092) together indicate that post-treatment roughness differences reflect differential surface modification patterns rather than pre-existing heterogeneity or abrasive tissue damage. The specific mechanism underlying this pattern, however, remains to be determined in studies with controlled surface contamination, standardized device-powder combinations, and surface composition analysis falling outside the scope of the present investigation.

Beyond average roughness, significant treatment effects were observed for maximum valley depth changes (ΔSv: F(3, 193) = 8.14, *p* < 0.001, η^2^*p* = 0.089) and peak height changes (ΔSp: F(3, 193) = 6.80, *p* < 0.001, η^2^*p* = 0.091). Erythritol generated the most pronounced increases in both parameters (ΔSv: 19.24 [0.96; 47.24] µm; ΔSp: 18.52 [0.84; 40.76] µm), significantly exceeding trehalose (*p* < 0.05). Post-treatment analysis confirmed that erythritol produced significantly higher Sv values compared to all other powders, while glycine and erythritol together produced significantly higher Sz values than trehalose. This dissociation between Sa and extreme height parameters (Sz, Sv) suggests that erythritol creates localized surface irregularities through focused impact events rather than uniform surface erosion - a pattern consistent with erythritol’s known kinetic properties and its small, dense particle structure [10]. Beyond particle size, hardness and density may additionally modulate the surface impact characteristics of the tested powders. Glycine and erythritol share a Mohs hardness of 2.0, while sodium bicarbonate is slightly harder at 2.5 [22]. In terms of bulk density, glycine (1.16 g/cm^3^ [23]), and erythritol (1.45 g/cm^3^ [24]), are considerably less dense than sodium bicarbonate (2.16 g/cm^3^ [25]), , resulting in lower kinetic energy per particle at equivalent velocity. Comparable physicochemical data for trehalose in the context of dental air-polishing powders are not consistently reported in the literature. These differences in physicochemical properties may contribute to the distinct surface modification patterns observed, though a systematic tribological characterization of all four powders falls outside the scope of the present study. If these topographical changes represented true surface destruction, corresponding substance loss would be expected; however, no significant differences in substance loss were detected between groups (*p* = 0.431, η^2^*p* = 0.017).

The Ssk data provide additional context for interpreting these findings. Although no significant between-group differences in post-treatment Ssk were detected (*p* > 0.05, η^2^*p* = 0.024), all powders showed a trend from positive toward negative Ssk values (ΔSsk: trehalose − 0.28 [-0.93; 0.22]; sodium bicarbonate − 0.46 [-1.31; 0.20]; erythritol − 0.17 [-1.40; 0.47]; glycine − 0.16 [-0.99; 0.29]). This shift from peak- to valley-dominated topography is consistent with the preferential modification of protruding surface features, potentially exposing the native root surface architecture though direct evidence for deposit removal was not obtained in this study. Natural root surfaces exhibit intrinsic negative skewness due to dentinal tubules and Sharpey fiber insertions [26], and the post-treatment profiles of erythritol and glycine most closely resembled this native morphology. Taken together with the Str findings (post-treatment values were borderline significant before FDR correction (*p* = 0.042), with erythritol producing more anisotropic patterns than trehalose and sodium bicarbonate) the data suggest that different powders engage the root surface through distinct mechanical mechanisms, even when overall substance loss is comparable. 

### Substance loss and cementum preservation

Increased surface roughness did not translate into greater substance loss. No significant differences in substance loss were detected between powder types (F(3, 193) = 0.92, *p* = 0.431, η^2^*p* = 0.017), with the maximum mean loss observed for trehalose at -13.0 ± 33.5 μm. The wide within-group standard deviations reflect the inherent biological heterogeneity of root surfaces, including substantial regional variation in cementum thickness [27]. Negative substance loss values, observed across all groups, are attributable to residual scan superimposition error rather than true tissue gain, as confirmed by a systematic measurement error of -0.7 μm in untreated control specimens. Notably, the maximum detectable substance change of -23.7 μm indicates that mean substance loss values for all groups approach or fall within the reliable detection limit of the system; absolute substance loss values should therefore be interpreted accordingly. The absence of significant between-group differences nonetheless confirms that no powder caused systematically greater cementum removal under the conditions tested, consistent with recent ex vivo data demonstrating low substance removal with low-abrasive air-polishing compared to conventional hand instrumentation [9]. The kinetic energy delivered by low-abrasive powders appears sufficient to modify the surface topographically while leaving the underlying cementum largely intact; a pattern not achievable with conventional curette or ultrasonic instrumentation, where force cannot be precisely controlled and over-instrumentation frequently occurs [28, 29]. Furthermore, dentin surfaces pretreated with erythritol airflow have been shown to exhibit greater resistance to subsequent chemomechanical challenge than surfaces treated with hand scalers or ultrasonics [10], suggesting a potential additional benefit beyond immediate debridement. Excessive cementum removal has been associated with compromised new attachment formation and increased risk of root sensitivity [30], underscoring the clinical importance of these topographic findings. 

### Clinical implications

These topographical findings provide a surface-level rationale for powder selection, though their clinical translation should be interpreted cautiously. The present study employed an ex vivo model without biofilm and cannot directly address debridement efficacy. Regarding surface roughness thresholds, a Ra of 0.2 μm has been proposed as a critical value for bacterial adhesion in the context of restorative materials [31, 32]; however, whether this threshold applies to root cementum and dentin under clinical conditions remains uncertain. The Sa values measured post-treatment in all groups substantially exceeded this threshold, reflecting the inherent roughness of root surfaces rather than a powder-specific risk. Clinically, a recent systematic review found comparable outcomes (probing depth reduction, clinical attachment gain, and bleeding on probing) across erythritol, glycine, and trehalose at 3 to 6 months [8], indicating that the surface roughness differences observed here do not necessarily translate into differential clinical effectiveness. Nevertheless, the distinct surface modification patterns observed under the present experimental conditions - particularly the greater exposure of native root topography and larger Sv/Sp values with erythritol and glycine - may be of interest during active periodontal therapy where thorough root surface debridement is the primary objective, though direct evidence for superior debridement efficacy is beyond the scope of the present study. Trehalose and sodium bicarbonate, producing smoother post-treatment surfaces, may be more appropriate during supportive therapy where preservation of surface characteristics is prioritized. All four powders demonstrated minimal and comparable substance loss, supporting their safety for repeated subgingival application on exposed root surfaces. 

### Limitations

The present study is subject to several limitations that may affect its generalizability and clinical transferability.

In particular, the use of non-validated powder-to-device connections represents a significant methodological constraint: The Prophyflex 4 device (KaVo, Biberach, Germany) used for all applications does not hold regulatory approval for all tested powders. Particle velocity, spray geometry, powder flowability, and aerosol dynamics are sensitive to the mechanical properties of the powder-to-device interface, including chamber design, nozzle geometry, and air pressure calibration [21]. Consequently, the observed differences in post-treatment surface roughness between powders may partially reflect device-to-powder interaction effects rather than, or in addition to, intrinsic powder abrasiveness. While all powders were tested under strictly identical bench-mounted conditions (3 mm distance, 45° angulation, 15 s application, maximum powder chamber fill), this standardization controls for operator variability but does not eliminate the possibility of differential powder behavior within the same device. These considerations limit the external validity of the absolute roughness values reported and should be taken into account when interpreting intergroup comparisons. Future studies should employ device-to-powder combinations with full regulatory approval and, where possible, manufacturer-validated delivery parameters to ensure that observed differences reflect powder properties rather than device compatibility effects. While this limitation affects absolute values, the relative comparisons between powders tested under identical conditions remain valid.

Further, the study employed handpieces mounted on a stand rather than full clinical delivery systems. Handpieces show a higher dependence on powder flow rate and air pressure distribution. To mitigate this concern, the powder chamber was filled to maximum capacity after each specimen. Nevertheless, this approach may yield different particle dynamics than those observed in clinical use with automated powder delivery systems. The air-polishing powders were only available in their commercially manufactured particle sizes, preventing standardization across all materials tested. This may have introduced bias when comparing surface roughness effects between different powder types. Second, specimens were stored for varying durations up to three months in Ringer’s solution following air-polishing treatment Although Ringer’s solution is a standard storage medium that preserves tissue integrity, variable storage durations may have introduced subtle differences in surface hydration and optical properties at the time of scanning. The paired pre- and post-treatment design and randomized group allocation mitigate, but do not fully eliminate, this potential source of bias. Future studies should aim to standardize the interval between treatment and final measurement.

Additionally, the relatively small measurement area (less than 1 mm^2^) may not capture the full heterogeneity of root surface characteristics across larger anatomical regions. Larger measurement fields would be desirable for a more representative topographical characterization; this must be balanced with the need to maintain precise focus on curved surfaces. Root surfaces exhibit substantial regional variation in surface topography depending on anatomical location and degree of previous disease activity [26]. The small measurement area enables high-resolution topographical analysis but may not capture the full extent of surface heterogeneity. Notably, the measurement error quantified by the control specimens was well within acceptable limits.

Furthermore, the high within-group variability in substance loss measurements reflects the inherent biological heterogeneity of root surfaces, including substantial regional differences in cementum thickness - ranging from 16 μm coronally to over 200 μm apically, and differing significantly between mesial and distal surfaces [27] - as well as prior disease-related surface alterations. This variability should be considered when interpreting absolute substance loss values, although it does not affect the validity of between-group comparisons.

The use of extracted teeth, while necessary for controlled laboratory investigations, cannot fully replicate the clinical environment. In vivo conditions include the presence of crevicular fluid, bleeding, soft tissue mobility, and limited visual and tactile access, all factors that influence the air-polishing technique and outcomes.

Finally, the study did not assess the biological consequences of the observed surface changes. While we can measure topographical alterations, their effects on subsequent biofilm formation, periodontal healing, and tissue regeneration require clinical investigation. Long-term clinical studies comparing healing outcomes following treatment with different powders would provide valuable complementary data. Furthermore, elemental surface composition was not assessed in the present study. Energy dispersive x-ray spectroscopy in combination with SEM would provide valuable complementary information on potential powder residues and compositional alterations to the cementum surface following air-polishing. Future studies should incorporate energy dispersive x-ray spectroscopy analysis to comprehensively characterize both the structural and elemental effects of low-abrasive air-polishing powders on root cementum.

## Conclusion

Erythritol and glycine powders produced more pronounced topographical changes on root surfaces compared to trehalose and sodium bicarbonate, which may reflect differences in surface interaction mechanisms rather than increased substance loss. The novel application of more detailed surface parameters, particularly Ssk, Sp, and Sv, enabled differentiation between distinct surface modification patterns across powders that are not captured with conventional amplitude parameters alone. Whether the observed topographical differences translate into clinically meaningful differences in debridement efficacy requires investigation in biofilm-containing models. 

## Electronic Supplementary Material

Below is the link to the electronic supplementary material.

Supplementary Material. Fig. S1: Three-dimensional laser scanning microscopy reconstructions of representative root surface specimens treated with the four tested powders: sodium bicarbonate (A, B), glycine (C, D), erythritol (E, F), and trehalose (G, H). Left column: before treatment; right column: after treatment. Laser scanning microscopy images were acquired at 10× magnification with a field of view of 800 µm along the x-axis and 250 µm along the y-axis.

Supplementary Material. Fig. S2: Boxplots showing all nine ISO 25178 surface roughness parameters: (A) ΔSa, arithmetical mean height; (B) ΔSz, maximum height; (C) ΔStr, texture aspect ratio; (D) ΔSpc, arithmetic mean peak curvature; (E) ΔSdr, developed interfacial area ratio; (F) ΔSsk, skewness; (G) ΔSp, maximum peak height; (H) ΔSv, maximum pit height; (I) ΔSal, auto-correlation length. Boxes represent the interquartile range (IQR) with the median shown as a horizontal line. Whiskers extend to 1.5 × IQR. Individual data points are displayed as grey dots. Different superscript letters indicate statistically significant differences between groups based on estimated marginal means adjusted for tooth type and surface (FDR-corrected p<0.05). Groups sharing the same letter are not significantly different.

Supplementary Material. Fig. S3: Box plots display mean ± standard deviation of substance change (µm) for each treatment group. Different superscript letters indicate statistically significant differences between groups based on estimated marginal means adjusted for tooth type and surface (FDR-corrected p<0.05). Groups sharing the same letter are not significantly different.

## Data Availability

No datasets were generated or analysed during the current study.
