# Supplementary material for: Comparative analysis of low-abrasive powders on root surface roughness and substance loss: an ex vivo study
Source: Clin Oral Investig. 2026 Jul 17;30(8):341. doi: 10.1007/s00784-026-07007-2 (PMC13379490; doi:10.1007/s00784-026-07007-2)
Supplement: Supplementary file 1 [file 784_2026_7007_MOESM1_ESM.odt]

Fig. SI: Three-dimensional laser scanning microscopy reconstructions of representative root surface specimens treated with the four tested powders: sodium bicarbonate (A, B), glycine (C, D), erythritol (E, F), and trehalose (G, H). Left column: before treatment; right column: after treatment. Laser scanning microscopy images were acquired at 10× magnification with a field of view of 800 µm along the x-axis and 250 µm along the y-axis.
