# Supplementary material for: Comparative analysis of low-abrasive powders on root surface roughness and substance loss: an ex vivo study
Source: Clin Oral Investig. 2026 Jul 17;30(8):341. doi: 10.1007/s00784-026-07007-2 (PMC13379490; doi:10.1007/s00784-026-07007-2)
Supplement: Supplementary file 2 [file 784_2026_7007_MOESM2_ESM.odt]

Fig. SII: Boxplots showing all nine ISO 25178 surface roughness parameters: (A) ΔSa, arithmetical mean height; (B) ΔSz, maximum height; (C) ΔStr, texture aspect ratio; (D) ΔSpc, arithmetic mean peak curvature; (E) ΔSdr, developed interfacial area ratio; (F) ΔSsk, skewness; (G) ΔSp, maximum peak height; (H) ΔSv, maximum pit height; (I) ΔSal, auto-correlation length. Boxes represent the interquartile range (IQR) with the median shown as a horizontal line. Whiskers extend to 1.5 × IQR. Individual data points are displayed as grey dots. Different superscript letters indicate statistically significant differences between groups based on estimated marginal means adjusted for tooth type and surface (FDR-corrected p<0.05). Groups sharing the same letter are not significantly different.
