# Supplementary material for: Comparative analysis of low-abrasive powders on root surface roughness and substance loss: an ex vivo study
Source: Clin Oral Investig. 2026 Jul 17;30(8):341. doi: 10.1007/s00784-026-07007-2 (PMC13379490; doi:10.1007/s00784-026-07007-2)
Supplement: Supplementary file 3 [file 784_2026_7007_MOESM3_ESM.odt]

Fig. SIII: Box plots display mean ± standard deviation of substance change (µm) for each treatment group. Different superscript letters indicate statistically significant differences between groups based on estimated marginal means adjusted for tooth type and surface (FDR-corrected p<0.05). Groups sharing the same letter are not significantly different.
